# Near-infrared germanium PIN-photodiodes with >1A/W responsivity

**DOI:** 10.1038/s41377-024-01670-4

**Published:** 2025-01-01

**Authors:** Hanchen Liu, Toni P. Pasanen, Tsun Hang Fung, Joonas Isometsä, Antti Haarahiltunen, Steven Hesse, Lutz Werner, Ville Vähänissi, Hele Savin

**Affiliations:** 1 Aalto University, Department of Electronics and Nanoengineering, Espoo, Finland; 2 ElFys, Inc., Espoo, Finland; 3 Physikalisch-Technische Bundesanstalt, Berlin, Germany

**Keywords:** Optoelectronic devices and components, Nanowires

## Abstract

Even though efficient near-infrared (NIR) detection is critical for numerous applications, state-of-the-art NIR detectors either suffer from limited capability of detecting incoming photons, i.e., have poor spectral responsivity, or are made of expensive group III-V non-CMOS compatible materials. Here we present a nanoengineered PIN-photodiode made of CMOS-compatible germanium (Ge) that achieves a verified external quantum efficiency (EQE) above 90% over a wide wavelength range (1.2–1.6 µm) at zero bias voltage at room temperature. For instance, at 1.55 µm, this corresponds to a responsivity of 1.15 A/W. In addition to the excellent spectral responsivity at NIR, the performance at visible and ultraviolet wavelengths remains high (EQE exceeds even 100% below 300 nm) resulting in an exceptionally wide spectral response range. The high performance is achieved by minimizing optical losses using surface nanostructures and electrical losses using both conformal atomic-layer-deposited aluminum oxide surface passivation and dielectric induced electric field -based carrier collection instead of conventional pn-junction. The dark current density of 76 µA/cm^2^ measured at a reverse bias of 5 V is lower than previously reported for Ge photodiodes. The presented results should have an immediate impact on the design and manufacturing of Ge photodiodes and NIR detection in general.

## Abstract (web summary)

A nanoengineered PIN-photodiode made of CMOS-compatible Ge achieves high external quantum efficiency over wide wavelength range (0.2–1.8 µm) at zero bias voltage at room temperature.

## Graphical abstract

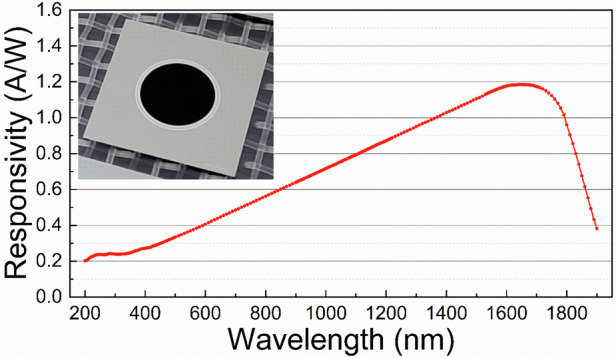

## Introduction

Nowadays remarkably many fields and technologies rely on near- and shortwave infrared (NIR, SWIR) radiation sensing including, e.g., healthcare, telecommunication, autonomous driving, agriculture, spectroscopy, and virtual reality – just to name a few^1–5^. The performance of the applications developed for these technologies is largely based on the ability to detect IR photons. Therefore, it is rather surprising that state-of-the-art NIR detectors are either made of very expensive and non-CMOS compatible materials or are able to detect only limited portion of incoming photons, i.e., their external quantum efficiency (EQE) is far from ideal. For instance, the highest performing photodiodes are able to detect roughly 80% of the photons at the most common telecommunication wavelength of 1.55 µm (at zero bias) but are made from the group III-V material indium gallium arsenide (InGaAs)^6,7^. InGaAs is expensive, hazardous to environment and health, and not CMOS compatible significantly complicating integration to final applications^8^. Recently, a less expensive alternative based on colloidal quantum dots has been developed, but it is able to convert only roughly 40% of the photons to collected electrons due to low absorption^9,10^. A third option is germanium (Ge), a group IV material similar to silicon but with a suitable band gap for NIR detection (up to 1.8 µm). Ge has potential to combine the benefits of both above mentioned technologies simultaneously, i.e., high performance, affordability, and scalability. Most importantly, Ge as a material is CMOS compatible, i.e., it can be in principle processed with the same process line than is used for CMOS electronics. Indeed, Ge-based photodiodes are already commercially available for a wide range of applications. Despite this, the reported performances are still far from ideal: The best performing Ge-based photodiodes are typically able to detect only 70% of the photons at 1.55 µm under zero bias, moreover, their quantum efficiency is strongly wavelength dependent^11,12^. In addition, they suffer from relatively high dark currents as the lowest reported values are in the 10^−4^−10^−3^ A/cm^2^ range^13–15^.

The modest performance of the state-of-the-art Ge photodiodes both in terms of absolute EQE and wavelength range can be explained by the fact that they still rely on rather traditional means for light management, carrier collection, and electrical passivation of surfaces. Regarding optics, single or multilayer antireflective coatings (ARC) are used to reduce the reflectance losses, but making them simultaneously effective over a broad wavelength range is challenging or even impossible^16^. Carrier collection is realized with conventional pn-junction using ion implantation that always introduces crystal damage and dopant-induced Auger recombination resulting in a so-called dead layer^17,18^. Furthermore, high diffusion of the dopant ions in Ge lattice further complicates the pn-junction formation in Ge devices^19^. Finally, as is well known, electrical passivation of Ge surfaces is challenging due to germanium oxide being highly unstable^20^. These difficulties deteriorate not only the quantum efficiency but also increase the dark current.

Recent advancements made in nanoengineering hold promise for enhancing the performance of Ge photodiodes. One such advancement is nanostructuring of the Ge surface, which has emerged as an intriguing alternative to ARCs^21,22^. The needle-like nanostructure forms a gradually changing refractive index at the Ge surface resulting in only ~1% reflectance across a wide spectral range (0.2–1.8 µm)^21^. Additionally, the nanostructures tend to scatter the incoming light possibly enhancing the absorption at long wavelengths^23^. However, the integration of nanostructures into actual Ge photodiodes is highly challenging because the issues encountered in planar devices regarding surface passivation and dead layer formation will be much more severe in nanostructures due to the significantly increased surface area^17^. Consequently, the traditional fabrication methods used for pn-junction formation and surface passivation cannot be used together with the nanostructures. Therefore, there is an obvious need to develop alternative approaches.

In this Article, we address the above challenges and propose a novel Ge photodiode concept for efficient NIR detection by integrating nanostructures into a Ge PIN photodiode. In order to make the integration successful and to avoid the dead layer formation, instead of conventional pn-junction formation, the core idea is to realize the carrier separation and subsequent collection via an electric field that is induced by a highly-charged dielectric layer deposited on top of the Ge nanostructures. We use atomic layer deposition (ALD) in order to obtain good conformality and surface passivation^24–27^. Keeping in mind the absorption coefficient of Ge (i.e. most photons absorb near the front surface), the elimination of dead layer should result in high performance. Indeed, we demonstrate unforeseen EQE over a broad wavelength range from 0.2–1.9 µm with zero bias voltage and without tradeoffs in dark current. The results are verified by independent measurements at PTB (Physikalisch-Technische Bundesanstalt).

## Results

### Detailed description of the photodiode structure

As mentioned above, in order to eliminate the reflectance losses, we need to fabricate nanostructures on the Ge photodiode surface. However, pn-junction formation in nanostructures via external doping is likely to result in a dead layer and low photoresponse. Therefore, we propose totally different means to form the electric field needed to separate and collect the photogenerated charge carriers. Instead of forming the field conventionally via external doping to form a pn-junction, we apply a dielectric layer with fixed charge on top of the Ge nanostructures to avoid any doping related recombination. If the fixed charge is of opposing polarity to the substrate doping, the surface layer starts to deplete from carriers leaving behind ionized bulk dopant atoms. Thus, the charge polarity changes at the dielectric/semiconductor interface inducing a strong electric field that has a maximum right at the Ge surface. (This is in contrary to pn-junction where the field peaks in the middle of the junction i.e. well below the surface.) Note that the strong electric field also induces a thin inversion layer under the dielectric. Consequently, the carrier distribution near the front surface resembles that of the conventional pn-junction, and hence, such configuration is sometimes in literature, in our opinion somewhat misleadingly, referred as an induced junction^28–30^.

**Fig. 1:**
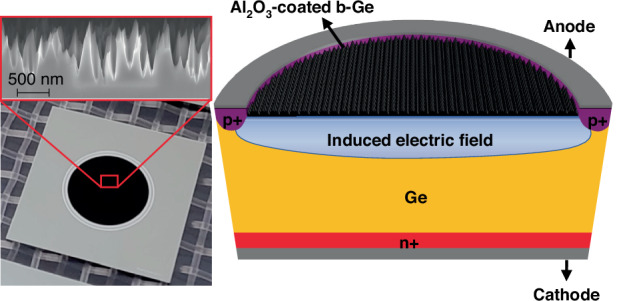
A concept for nanostructured Ge photodiode. The figure presents a photograph and a cross-sectional schematic of a 5-mm-diameter nanostructured Ge photodiode fabricated in this work. The inset shows a high magnification cross-section SEM image of the nanostructured surface that covers the whole active area and is seen black to the naked eye. The diode has an electric field right at the surface that is induced by the negatively-charged Al2O3 thin film

Figure 1 shows a cross-sectional schematic of our photodiode concept combining nanostructured Ge surface, ALD aluminum oxide (Al_2_O_3_) surface passivation, and negatively charged Al_2_O_3_- induced electric field for carrier separation and hole collection. Additionally, a photograph of the final device fabricated in this work and an SEM image of the Ge surface nanostructures are shown. The starting material was n-type Czochralski-grown Ge wafer with a resistivity of 29.1 ± 4.8 Ωcm, a thickness of 302 µm, and a bulk lifetime of over 7 ms. The nanostructures were fabricated on the active area via cryogenic reactive ion etching process described in ref. ^21^. ALD Al_2_O_3_, with a measured charge density of -2.3×10^12 ^cm^−2^, was deposited on top of the nanostructures^24,26^. Ohmic contacts for cathode and anode were prepared using ion implantation followed by aluminum (Al) sputtering. A more detailed sample preparation process is provided in the Materials and methods section.

### Spectral responsivity at 0.2–1.9 µm

**Fig. 2:**
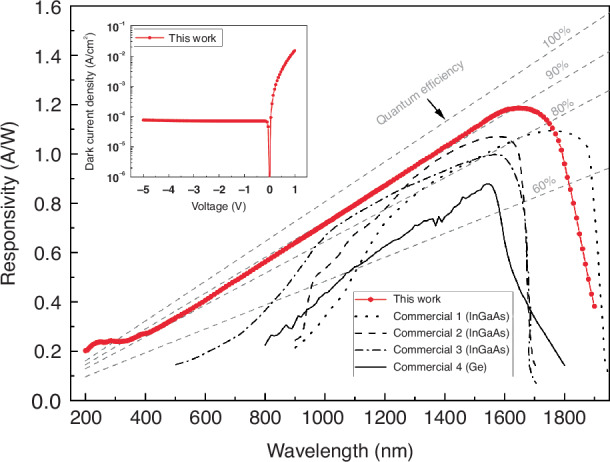
Measured spectral responsivity and comparison to literature The spectral responsivity of 5-mm-diameter nanostructured Ge photodiode fabricated in this work (red line) is shown as a function of wavelength measured at zero bias. The measurement uncertainty (provided separately for each wavelength in Supplementary information) varies within 0.2–3.4%. The gray dashed lines indicate the spectral responsivity of photodiodes with 60–100% EQE. The photodiodes with the highest spectral responsivity found from literature are shown as reference: 1, 2 and 3 are InGaAs photodiodes^31^, and 4 is a Ge photodiode^32^. The inset shows the dark current density measured from the nanostructured Ge photodiode

The ability of our Ge photodiode to convert photon energy into current as a function of wavelength is shown in Fig. 2 as spectral responsivity (measurement certificate is provided as Supplementary information). For a very wide range of wavelengths all the way from ultraviolet (UV, 0.2 µm) to NIR (1.7 µm), our photodiode is able to detect more than 80% of the incoming photons (see the Materials and methods section for the equation used to determine EQE from the spectral responsivity data). From the scientific point of view, it is very interesting to note that at UV, even above 100% EQE is reached. To our knowledge above 100% EQE has not been observed in Ge photodiodes before due to the fact that UV photons are usually absorbed within the dead layer just below the surface. This result will be analyzed in more detail later on together with internal quantum efficiency (IQE). The peak responsivity of our photodiode is as high as 1.19 A/W at 1.65 µm. The responsivity at wavelengths relevant for telecommunication applications (1.3 µm and 1.55 µm) is 0.95 A/W and 1.15 A/W, respectively. Even at 1.9 µm the responsivity remains reasonably high (0.38 A/W).

For reference devices we have selected the best-performing InGaAs photodiodes optimized for three different wavelengths and one best-performing Ge photodiode^31,32^ (shown also in Fig. 2). Surprisingly, our Ge photodiode outperforms all the InGaAs photodiodes both in terms of absolute EQE at zero bias at their peak wavelengths as well as in the broadness of the spectral range. It is good to note that the bandgap of InGaAs can be tailored to allow detection of wavelengths all the way to 2.5 µm although this comes at the expense of lower EQE at shorter wavelengths. Table 1 presents quantitative EQE, responsivity and detectivity for three selected wavelengths (1.3 µm, 1.55 µm, and 1.75 µm) for both our and the reference photodiodes. In our Ge photodiode, the responsivity at all three wavelengths is clearly higher than in InGaAs references. As expected, the performance of the Ge reference photodiode is clearly behind our device. For instance, the EQE and the peak responsivity at 1.55 µm are 68% and 0.85 A/W, respectively. The fact that the Ge photodiode fabricated here outperforms even the InGaAs photodiodes demonstrates the potential of our novel concept for boosting spectral responsivity.

**Table 1:** Comparison of *EQE*, corresponding responsivity (*R*) and detectivity (D*) at three selected wavelengths between this work and previous literature including the best-performing InGaAs and Ge photodiodes reported so far

Photodiode	*EQE* [%]/*R* [A/W]/D* [cmHz^1/2^/W]		
	λ_1_ = 1300 nm	λ_2_ = 1550 nm	λ_3_ = 1750 nm
**This work (Ge)**	**90.6%/0.95/1.41 × 10**^**11**^	**92.0%/1.15/1.71 × 10**^**11**^	**79.4%/1.12/1.67 × 10**^**11**^
Commercial 1 (InGaAs) Hamamatsu G12181_020K	79.2%/0.83/8.15 × 10^11^	88.0%/1.10/1.08 × 10^12^	77.9%/1.10/1.08 × 10^12^
Commercial 2 (InGaAs) Hamamatsu G8370-85	86.8%/0.91/3.47 × 10^12^	88.0%/1.10/4.19 × 10^12^	-/-/-
Commercial 3 (InGaAs) Hamamatsu G10899-03K	83.9%/0.88/2.91 × 10^12^	80.0%/1.00/3.31 × 10^12^	-/-/-
Commercial 4 (Ge) Thorlabs FDG50	67.7%/0.71/6.51 × 10^10^	68.0%/0.85/7.79 × 10^10^	17.0%/0.24/2.20 × 10^10^

The spectral responsivity alone does not determine the sensitivity of the photodiode. Another important parameter is the dark current, which is the photodiode current without any illumination. The dark current and its noise set the lower limit for the detectable light intensity. Therefore, we fabricated an additional batch of photodiodes including a guard ring structure to reduce the dark current. The inset of Fig. 2 shows an example of I-V characteristics of our Ge photodiode measured from this additional batch. The dark current is 350 nA at 1 mV reverse bias and increases to 15 µA at 5 V. Since the photodiode active area is circular with a diameter of 5 mm, the corresponding dark current densities are 1.8 µA/cm^2^ and 76 µA/cm^2^, respectively. These values are comparable or even below the best values reported earlier for bulk Ge photodiodes (100–1000 µA/cm^2^)^13^. The dark current in the best commercial Ge photodiodes is also at the same level (e.g., at 5 V, Teledyne J16~254.6 µA/cm^2^, Thorlabs FDG50 ~ 305.6 µA/cm^2^ with the corresponding photodiode area^32,33^). The low dark current obtained in our photodiode is a somewhat surprising result since while the device active area remains the same, the surface nanostructures make the actual physical surface area in our photodiode much (~7 times) larger. Larger surface area is known to increase the effective interface defect density increasing carrier generation and should thus increase the dark current as well. On the other hand, we use ALD Al_2_O_3_ surface passivation, which based on recent literature results in good surface passivation also on nanostructured Ge surfaces provided that sulfur and fluoride residues resulting from plasma etching have been completely removed^26^. Additionally, it is reported that a special chemical pre-treatment and a post-deposition anneal are also required to form a high quality interface between Ge and Al_2_O_3_^24,34,35^. Note that while the spectral responsivity of our Ge photodiode was higher than for InGaAs photodiodes, the dark currents are not comparable at room temperature due to significantly higher intrinsic carrier concentration ( ~ 10^13 ^cm^−3^ Ge vs. ~10^11 ^cm^−3^ InGaAs).

The spectral responsivity and the dark current together with other noise components such as Johnson and flicker noise determine the so-called specific detectivity D* that describes the capability of the photodiode to detect small light intensities. The specific detectivity of our photodiode at the three selected wavelengths and with the bias voltage of -5V are shown in Table 1. These values were calculated from zero bias Noise Equivalent Power and spectral responsivity with equation described in more detail in the Materials and methods section. The specific detectivity of our photodiode at the wavelength of peak responsivity (1650 nm) is 1.77 × 10^11^ Jones. While it is clear that our Ge photodiode cannot outperform InGaAs devices due to the above-mentioned significant difference in dark current, the detectivity of our photodiodes outperforms clearly the reference Ge device.

#### Optical properties

**Fig. 3:**
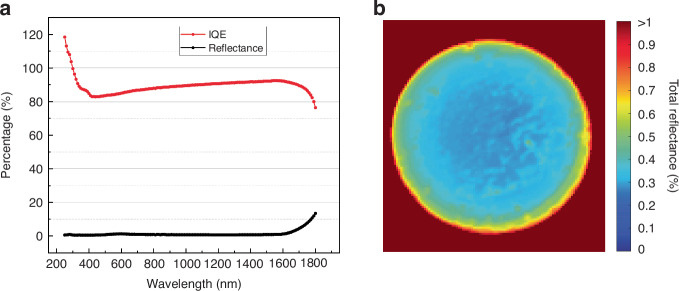
Measured reflectance and internal quantum efficiency. **a** The total reflectance (black) and the internal quantum efficiency (red) of the nanostructured Ge photodiode as a function of wavelength. **b** A reflectance map of a 5-mm-diameter nanostructured Ge photodiode measured at a wavelength of 656 nm

In order to study in detail the root causes for the above demonstrated high performance, we first characterized the spectral reflectance as well as the spatial reflectance distribution of the nanostructured photodiode. Figure 3a shows that the nanostructured surface has a very low reflectance (<1%) from UV all the way to NIR. At ~1600 nm, where the absorption depth in Ge increases steeply, the reflection from the wafer back surface starts to contribute and is seen as increased reflectance. Such a low reflectance is typical for nanostructured surfaces and is explained by a graded refractive-index interface present in high aspect ratio nanostructures with dimensions in the same range as the wavelength of incoming light and is studied extensively with optical simulations^36–38^. The measurement result confirms that we have been able to eliminate the reflectance losses. The spatial reflectance map (Fig. 3b) confirms that the low reflectance is obtained over the whole diode active area. Spatial uniformity is especially important in image sensors^39^ but also in single-pixel photodiodes^40^.

#### Internal collection efficiency

After measuring both spectral responsivity and reflectance, it is straightforward to determine the IQE, which describes the internal recombination losses (Fig. 3a). We can see that the IQE remains high (~90%) and relatively flat from 420 nm to 1700 nm. In NIR the highest IQE (~92.5%) is measured at around 1.55 µm. While the nanostructures are likely to somewhat increase the optical path length, in practice, after 1700 nm the IQE starts to decrease due to the increased absorption length extending beyond the used wafer thickness. Regarding deep UV region, as already indicated by spectral responsivity, it is notable that IQE starts to increase rapidly below 420 nm so that after 300 nm it is already above 100%. This indicates that one photon is able to create more than one electron. Above unity IQE (and EQE) has been observed earlier both in silicon solar cells^41^ as well as in photodiodes^30,42^. The underlying physics was studied in more detail in ref.^43^. All the reported results consistently showed that in silicon this was based on multiple carrier generation by impact ionization, i.e., a single high-energy UV photon produced high-energy carrier(s), which then generated further carriers^44^. Similar phenomenon is likely to be present in germanium as well. Another possibility is that UV photons have enough energy to directly generate several charge-carrier pairs. Historical studies on internal photoelectric effect in Ge indicate that the onset of multiple carrier generation should take place at around 460-575 nm^45,46^. Note that carrier multiplication has not been observed earlier in Ge devices without external bias voltage due to the losses related to pn-junction, surface recombination and reflectance.

#### Electric field distribution and conductive layer formation

Although the IQE is relatively flat and remains high in general over a wide wavelength range, a closer look reveals that it falls below 90% at 1000 nm and experiences a constant decrease until 440 nm where it reaches its local minimum value of 82.8%. Within this wavelength regime, absorption depth is below 800 nm. Keeping in mind that the nanostructure height is roughly 700 nm, it means that all the photons with a wavelength below 1000 nm are in practice absorbed inside the nanostructures.

**Fig. 4:**
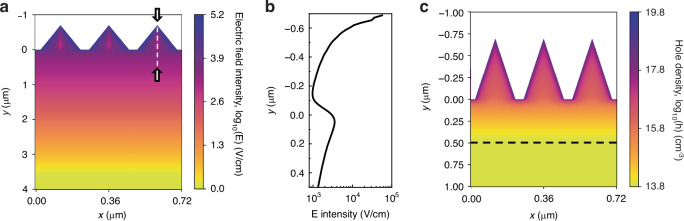
Simulated electric field and carrier concentrations in the nanostructures. **a** Simulated electric field distribution in our Ge photodiode demonstrating that the field penetrates deep into the substrate. The dashed white line shows the line scan position for (**b**). **b** A line scan of the electric field from the tip of the nanostructure towards the bulk. **c** Simulated hole concentration inside the nanostructures demonstrating the formation of a laterally conductive layer at the surface. The inversion layer edge is shown as a black dotted line

To shed more light on the IQE behavior, we simulate the electric field distribution in the nanostructures (Fig. 4a). The electric field extends well below the nanostructures all the way into the depth of ~4 µm. Thus, the reason for the decrease seen in IQE is not the fact that the absorption would take place outside the electric field. On the other hand, the electric field strength decreases exponentially towards the bulk, which could cause a reduction in IQE with increasing wavelengths. However, here we actually observe the opposite wavelength dependency. A closer look at the electric field inside a single needle reveals that in the needle the electric field is strongly concentrated in the vicinity of the surface and follows the nanostructure morphology (Fig. 4b). When moving from the surface towards the center of the needle, the field starts to diminish quickly and a local electric field minimum forms in the middle of the needle. Below the local minimum, the electric field starts to increase again before fading away deep in the bulk. Although not likely, we cannot rule out the possibility that this local electric field minimum would contribute to the local minimum of IQE at around 440 nm. The more probable reason for reduced IQE is, however, the higher surface recombination velocity (S_eff_) in nanostructured Ge than in planar surfaces even with high-quality ALD Al_2_O_3_ surface passivation^26^. Note that the surface passivation in our device is still quite good (S_eff_ of 38.5 cm/s) leading to high IQE also at short wavelengths.

In addition to the electric field, it is interesting to study carrier concentration inside the nanostructures. In a conventional PIN photodiode, the dopant density is on purpose made relatively high (via diffusion or implantation) close to the front surface to transport collected carriers laterally. Namely, a charge-conducting layer is always present. In the case of an electric field induced by the charge in the dielectric, this is not always the case. Only if the charge is large enough, a conductive inversion layer forms to the vicinity of the surface. As the simulations (Fig. 4c) show, in our device the charge density of the Al_2_O_3_ (-2.3 × 10^12^ cm^−2^) is high enough to result in a hole concentration that is clearly larger than the background electron concentration. Consequently, an inversion layer is formed at the surface. This inversion layer itself does not separate the carriers but in the actual device, it is important as the holes need to move laterally to the contacts.

## Discussion

The sensitivity of the photodiode is determined by the combination of the spectral responsivity and the dark current. The spectral responsivity of our device is already quite close to ideal, i.e., we are able to detect ~90% of the photons in a wide wavelength range. For NIR region there is no clear way to improve the response further, while in UV-Vis, more efficient utilization of carrier multiplication could result in above unity IQE already at ~550 nm instead of ~300 nm reported here. In our device, S_eff_ (38.5 cm/s) is much higher than has been achieved for planar Ge surfaces (6.6 cm/s). If such a low value could be achieved for the nanostructured Ge surface, we believe the positive impact of carrier multiplication would be seen at longer wavelengths. Regarding the dark current, we reported here values that are comparable (or lower) than has been reported earlier for Ge photodiodes. Despite that, we believe that the dark current can still be reduced further. For instance, we have not optimized contact implantations or substrate doping. Moreover, the above-mentioned S_eff_ of 38.5 cm/s is likely a significant source for the dark current. Naturally, there are also fundamental limitations for Ge dark current originating from the high intrinsic carrier concentration causing thermal noise at room temperature, but this can be mitigated via liquid nitrogen cooling.

We expect that the novel Ge photodiode concept presented here can be directly implemented to various kind of applications and, consequently, to improve the sensitivity. The fabrication methods that we present here should be readily available in all photodiode manufacturing facilities. The only concern is related to the etching of nanostructured Ge and related Ge thickness consumption, i.e., if the photodiodes are made on thin epitaxial Ge layers (typically only a few microns thick to avoid dislocations), one needs to develop the etching process not to consume epilayer too much. Furthermore, it is good to keep in mind that the quality of the epilayer might not be as high as for the CZ-wafers used here that might affect the final device performance. Another example where the new concept could be utilized is a NIR CMOS-image sensor similarly as has been reported for nanostructured silicon for visible wavelengths earlier^39^.

In summary, we have presented a novel Ge photodiode concept for NIR detection, which omits the need for pn-junction formation and antireflection coatings. We demonstrated its potential by fabricating a proof-of-concept device that achieved a record-high photoresponsivity (and EQE between 83%-125%) over a wide wavelength range (200 nm to 1700 nm) combined with a low dark current (76 µA/cm^2^ at 5 V). The high performance was found to originate from (i) minimal reflection losses enabled by surface nanostructures, (ii) high internal collection efficiency provided by the dielectric-induced electric field concentrated to the immediate vicinity of the surface, and (iii) high quality surface passivation obtained via a conformal ALD Al_2_O_3_ coating. The presented concept should be directly applicable to the manufacturing of state-of-the-art Ge photodiodes and thus paves the way for better performing NIR applications.

## Materials and methods

The photodiodes were fabricated on 100-mm-diameter, 302 µm thick single-side polished n-type (antimony doping) Ge wafers with <100> orientation. The resistivity was 24.2-33.9 Ωcm, as measured from the ingot by the wafer manufacturer. First, a 300 nm silicon nitride (SiN_*x*_) implantation mask was deposited on the front surface via plasma-enhanced chemical vapor deposition (PECVD) and patterned via standard photolithography and buffered hydrofluoric acid (BHF). The front side was implanted with boron (30 keV, 10^15^ cm^−3^) to form a heavily-doped p-type region around the active area, which enables ohmic contact formation with aluminum. The rear side was implanted with phosphorus (60 keV, 10^15^ cm^−3^) without a mask to form a highly-doped n^+^ layer for the same purpose. Next, the wafers were annealed at 500 °C in nitrogen (N_2_) ambient for 5 min to activate the implanted dopants. (Note that lower temperatures, around 400 °C, can also work for ohmic contact formation if smaller thermal budget is desired.) After removing the implantation mask in BHF, ALD Al_2_O_3_ etch mask was deposited on the front surface, followed by opening of the active area with a diameter of 5 mm. Nanostructures were then fabricated on the active area via inductively coupled plasma reactive ion etching (ICP-RIE) using a process reported by Pasanen et al. ^21^. An SEM image of the resulting nm-scale surface structure is presented in Fig. 1. The nanostructures were etched back in 3% v/v hydrogen peroxide (H_2_O_2_) for 15 s to optimize the balance between reflectance and surface passivation^26^. The Al_2_O_3_ etch mask was removed in BHF, followed by a dip in 31.6% v/v HCl for 5 min and the deposition of another 20 nm Al_2_O_3_ thin film via thermal ALD on the substrate front surface. The Al_2_O_3_ film was opened from the contact locations and annealed at 400 °C in N_2_ for 30 min to activate efficient surface passivation and to induce the electric field. 300 nm of Al was sputtered on the front surface to form the anode contact and patterned by lift-off to cover only the boron-doped ring-shaped pattern (Fig. 1). Similar Al layer was sputtered on the whole rear surface to form the n-type contact. The wafers were finally annealed at 350 °C in N_2_ for 30 min to heal possible sputtering-induced damage in the Al_2_O_3_ film^47^.

The spectral responsivity of the photodiode was determined by Physikalisch-Technische Bundesanstalt (PTB) by comparison with secondary detector standards using a measurement facility consisting of a radiation source, a double-grating monochromator with order-sorting filters, and a detector positing system. A xenon short-arc lamp and a tungsten-filament halogen bulb lamp were used as radiation sources in the wavelength ranges below 410 nm and from 390 nm to 1900 nm, respectively. The spectral responsivity of the secondary detector standards had previously been calibrated against PTB’s primary national detector standards for optical radiant power, the cryogenic electrical substitution radiometers. A monochromator-based cryogenic radiometer calibration facility was used in the UV spectral range from 200 nm to 410 nm and in the near infrared from 950 nm to 1900 nm whereas a laser-based cryogenic radiometer calibration facility was used in the wavelength range from 360 nm to 1005 nm. The official report of the certified measurements is included in the Supplementary information.

External quantum efficiency *EQE*(*λ*) was calculated from the measured spectral responsivity ${R}_{\lambda }$ via

$${EQE}\left(\lambda \right)=\frac{{R}_{\lambda }}{\left(\frac{q\lambda }{{hc}}\right)}$$

where $q$ is the elementary charge, *h* is the Planck constant, *c* is the speed of light in vacuum, and *λ* is the vacuum wavelength.

Specific detectivity (D*) was calculated with refs. ^48^ and ^49^ via

$${D}^{* }=\frac{\sqrt{A}{R}_{\lambda }}{\sqrt{2q{I}_{d}+\frac{4{kT}}{{R}_{0}}}}$$

where $A$ is the surface area of the detector, $k$ is the Boltzmann constant, $T$ is the temperature, ${I}_{d}$ is the dark current under specified reverse bias and *R*_*0*_ is the shunt resistance (calculated under ±10 mV by Ohm’s law). We expect the dark current along with Johnson noise to be the dominating noise sources when our photodiode is reverse biased and hence other noise sources, such 1/f (flicker) noise, are neglected in the calculation.

Total reflectance spectra of nanostructured surfaces were characterized from separate samples with an integrating sphere-based setup. These samples were of the same substrate material and experienced the same surface treatments (including nanostructure fabrication, H_2_O_2_ etch back, and ALD Al_2_O_3_ coating) as the photodiodes. Additionally, to investigate uniformity of the surface reflectivity, reflectance map was measured over the 5-mm-diameter active area of the finished photodiode (PV-2000, Semilab). Current-voltage (I-V) characteristics of the photodiodes were measured in the dark with a semiconductor parameter analyzer (Agilent/HP 4155C).

Carrier profiles and electric field near the front surface were simulated using the commercial software Silvaco Atlas. The Ge nanostructure dimensions were as follows: 200 nm width and 700 nm height. Nanostructures had conformal 20 nm thick Al_2_O_3_ layer on top. The fixed charge density of Ge/Al_2_O_3_ interface was set to −2.3 × 10^12^ cm^−2^. Bulk n-type doping concentration was set to 7 × 10^13^ cm^−3^.

## Supplementary information

Supplemental material

## Data Availability

The data that support the findings of this study are available from the corresponding authors on reasonable request. They are also available at figshare: 10.6084/m9.figshare.27612429.
