# Supplementary material for: Near-infrared germanium PIN-photodiodes with >1A/W responsivity
Source: Light Sci Appl. 2025 Jan 1;14:9. doi: 10.1038/s41377-024-01670-4 (PMC11688423; doi:10.1038/s41377-024-01670-4)

Supplementary Material for

**Near-infrared germanium PIN-photodiodes with >1 A/W responsivity**

Hanchen Liu^1^, Toni P. Pasanen^1,2^, Tsun Hang Fung^1^, Joonas Isometsä^1^, Antti Haarahiltunen^2^, Steven Hesse^3^, Lutz Werner^3^, Ville Vähänissi^1^ and Hele Savin^1^

^1^ Aalto University, Department of Electronics and Nanoengineering, Tietotie 3, 02150 Espoo,
 Finland
^2^ ElFys, Inc. Tekniikantie 12, 02150 Espoo, Finland

^3^ Physikalisch-Technische Bundesanstalt, Abbestraße 2–12, 10587 Berlin, Germany

1. Measurement certificate from PTB (Physikalisch-Technische Bundesanstalt).


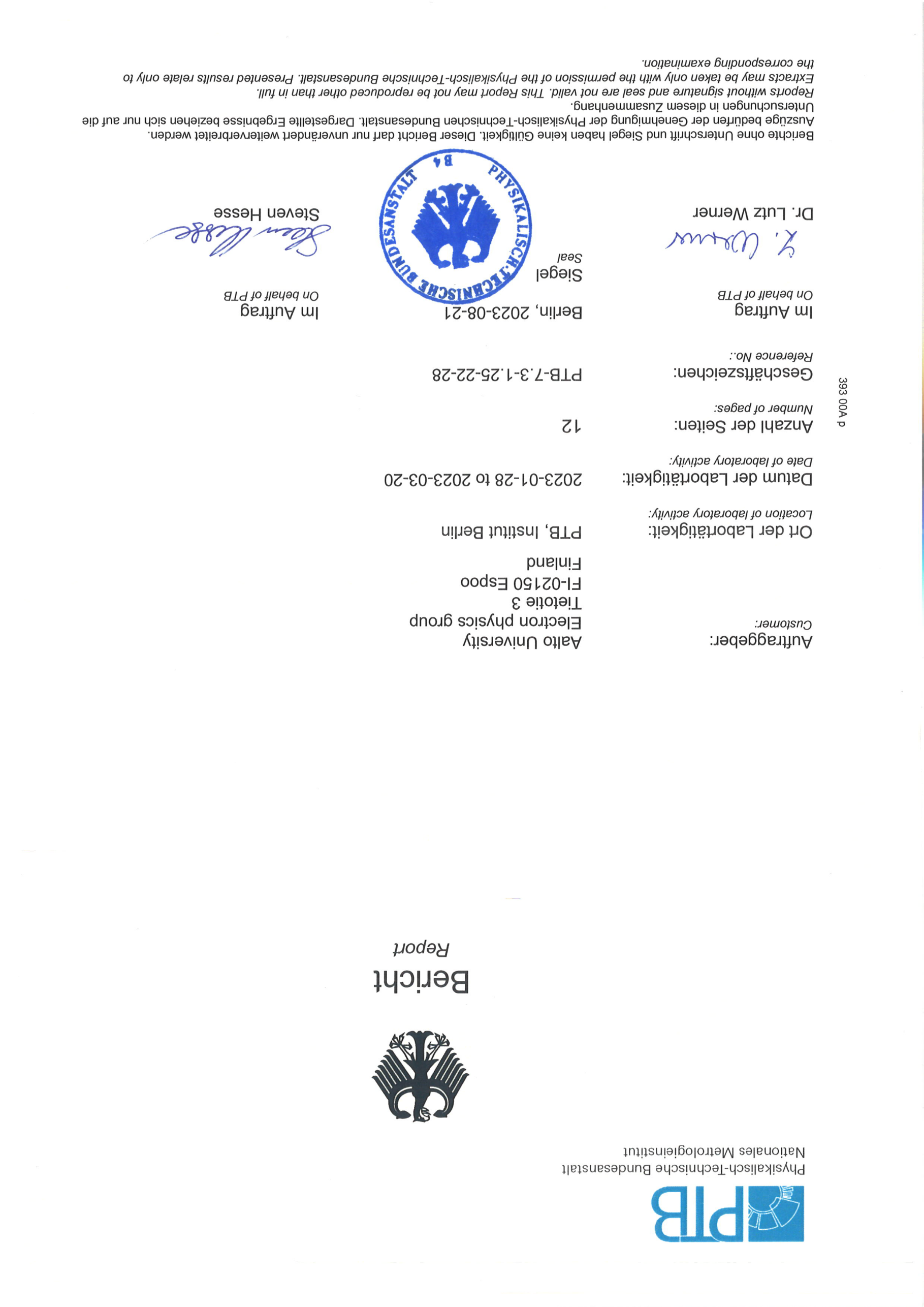


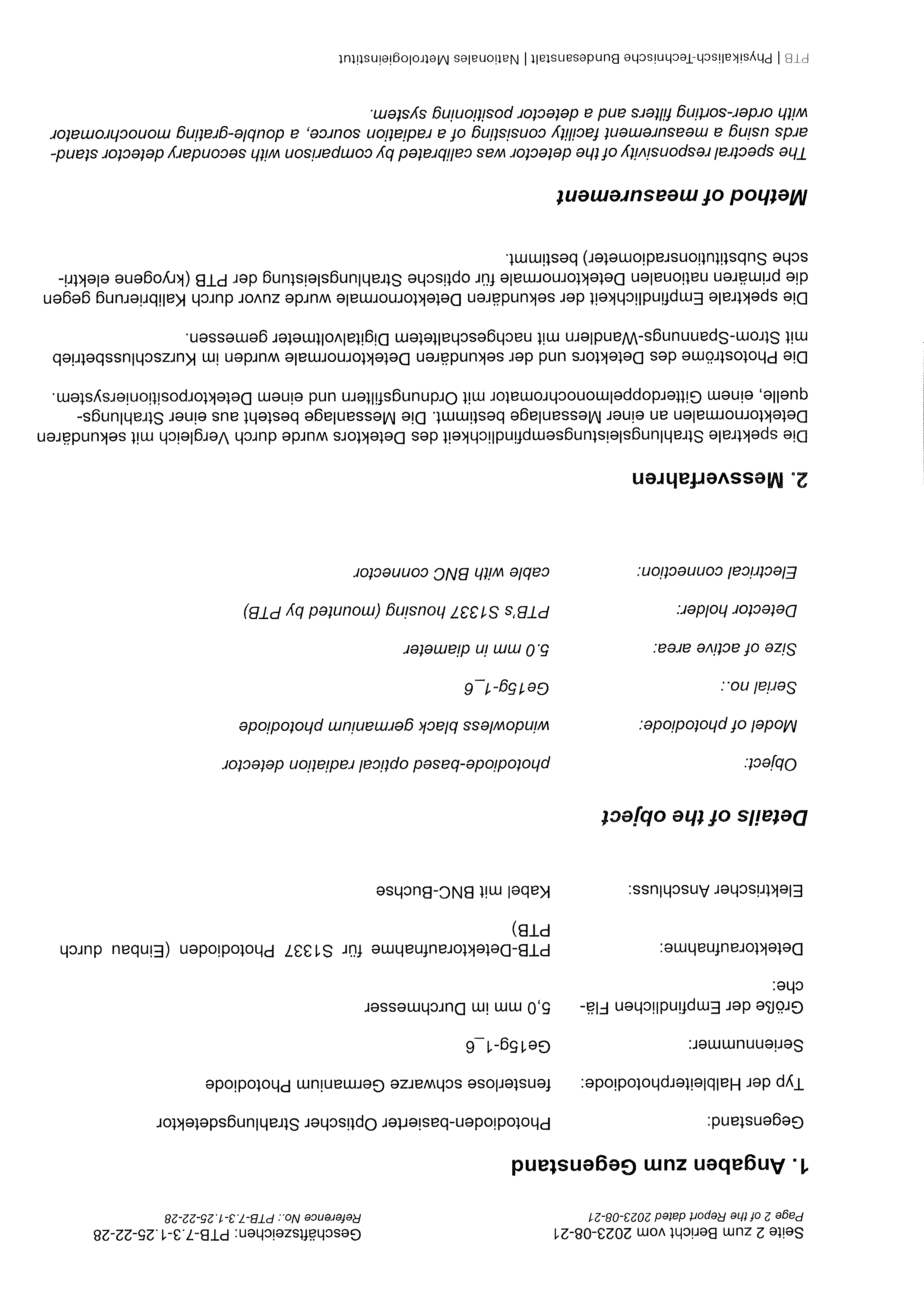


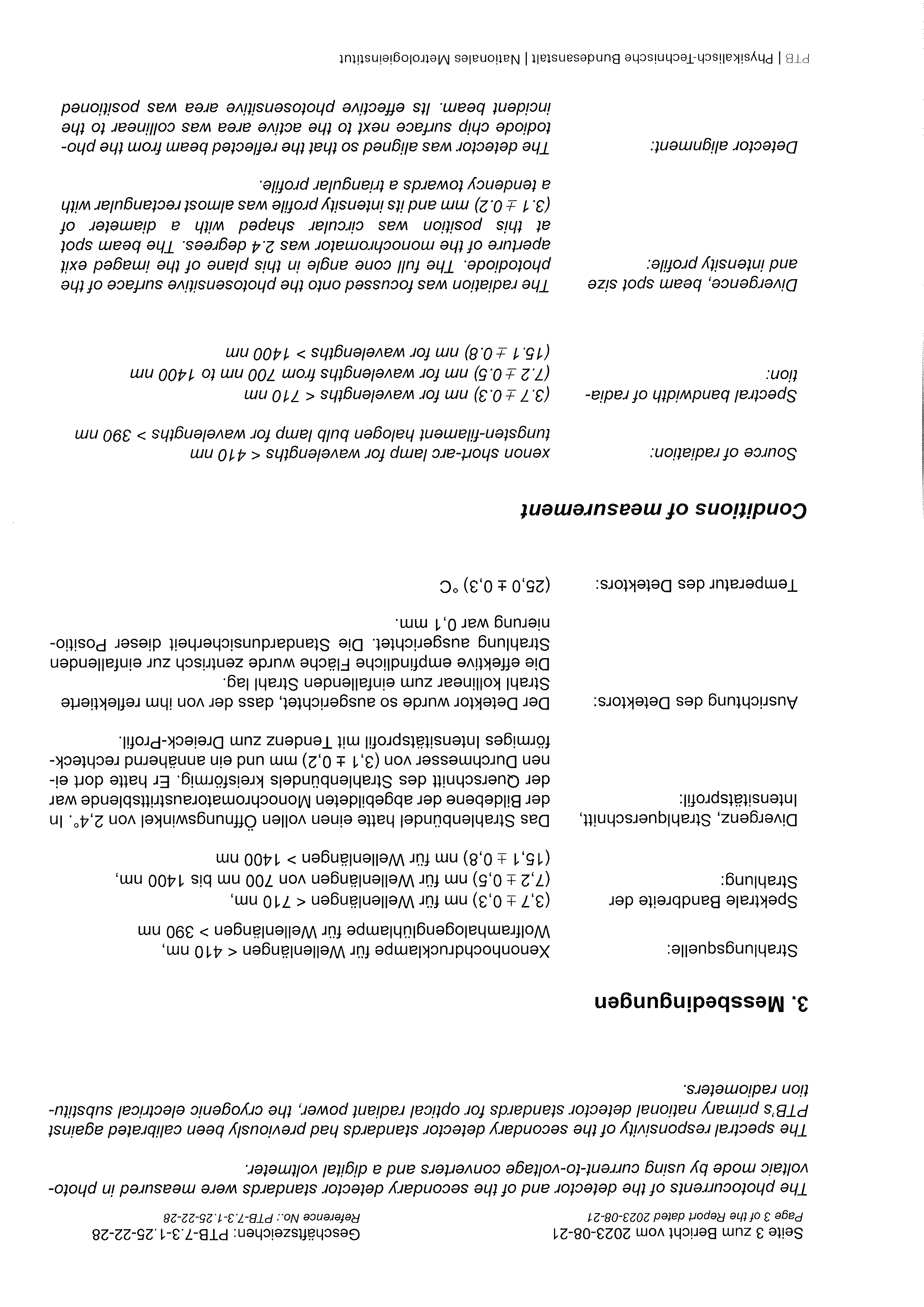


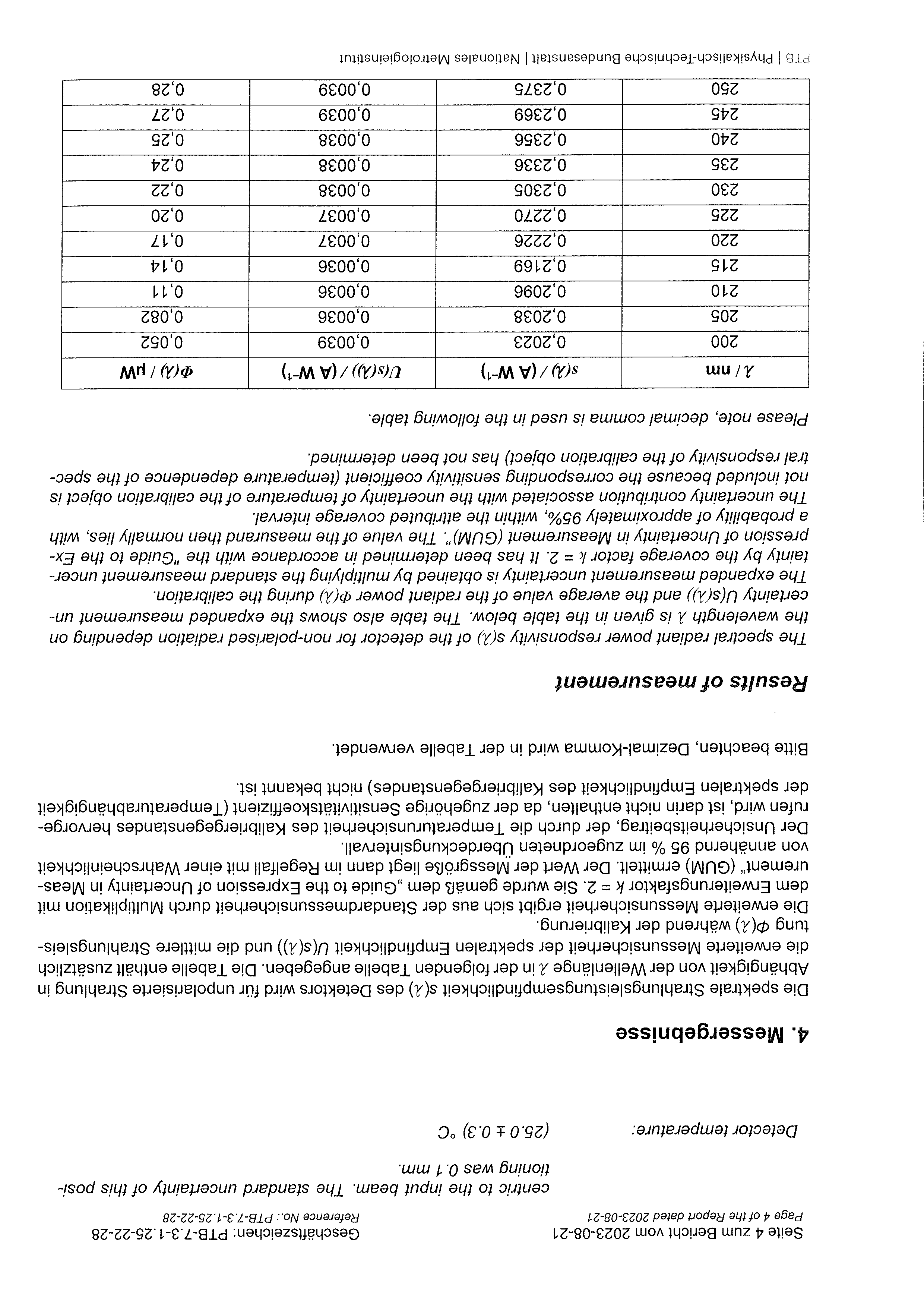


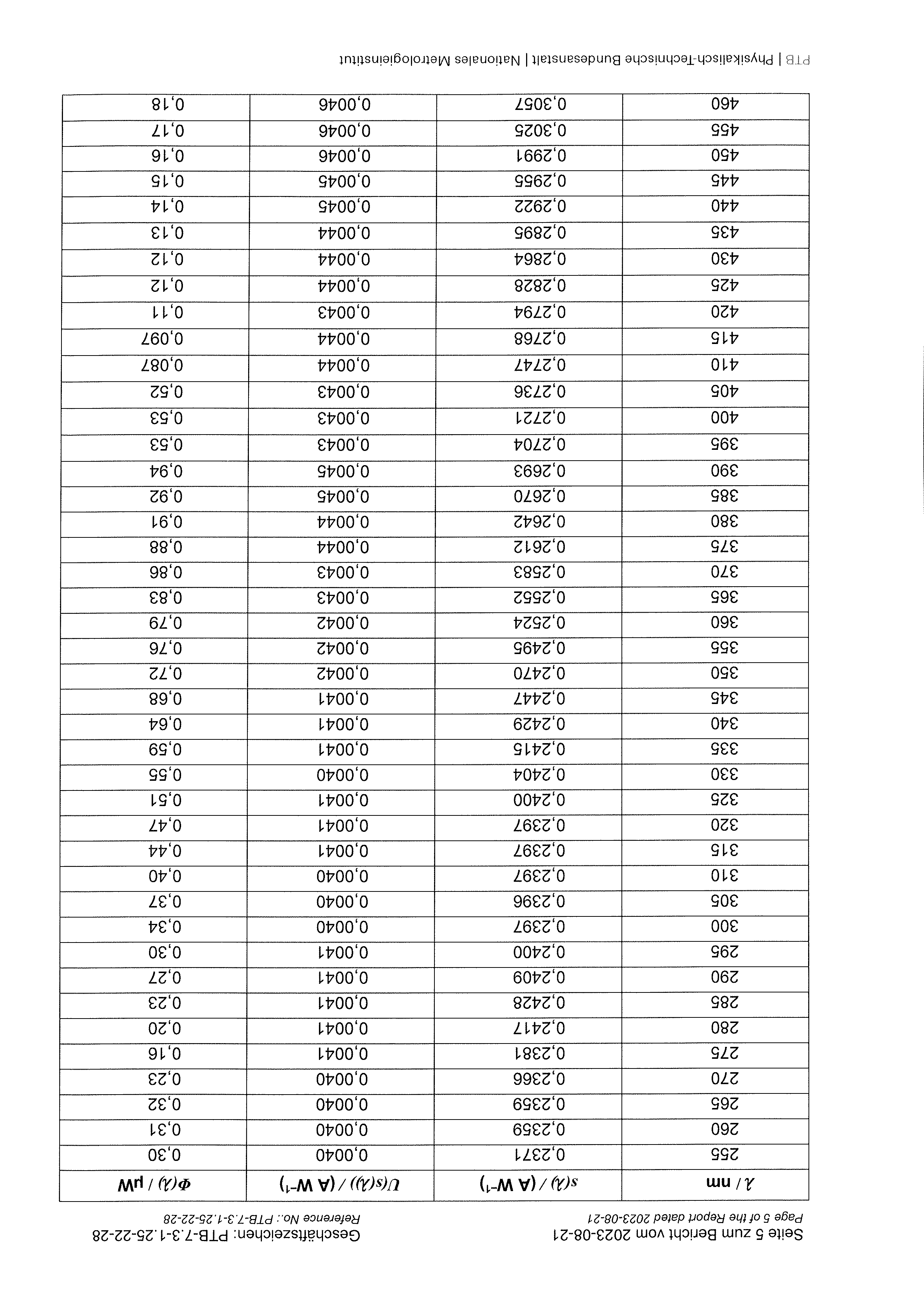


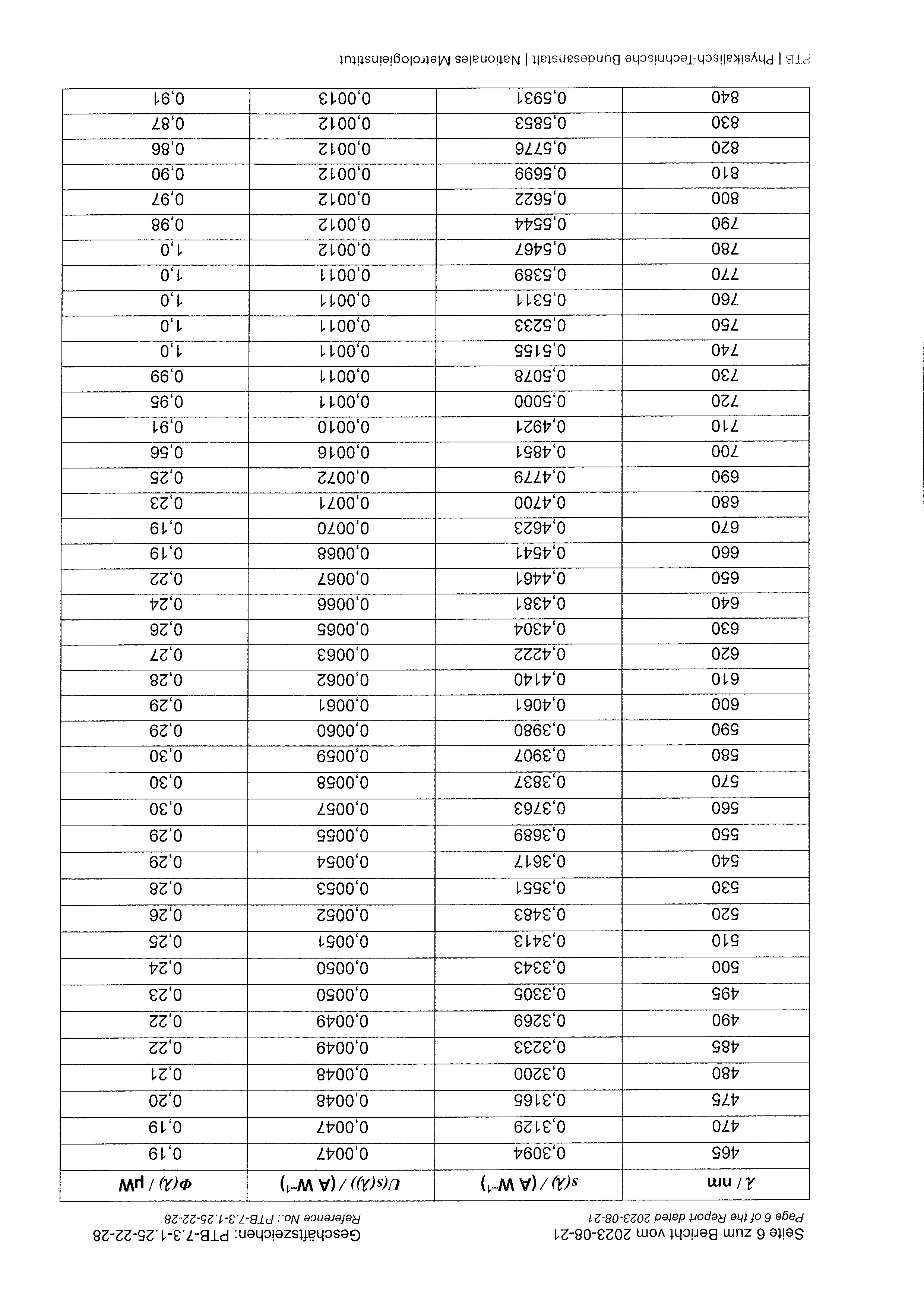


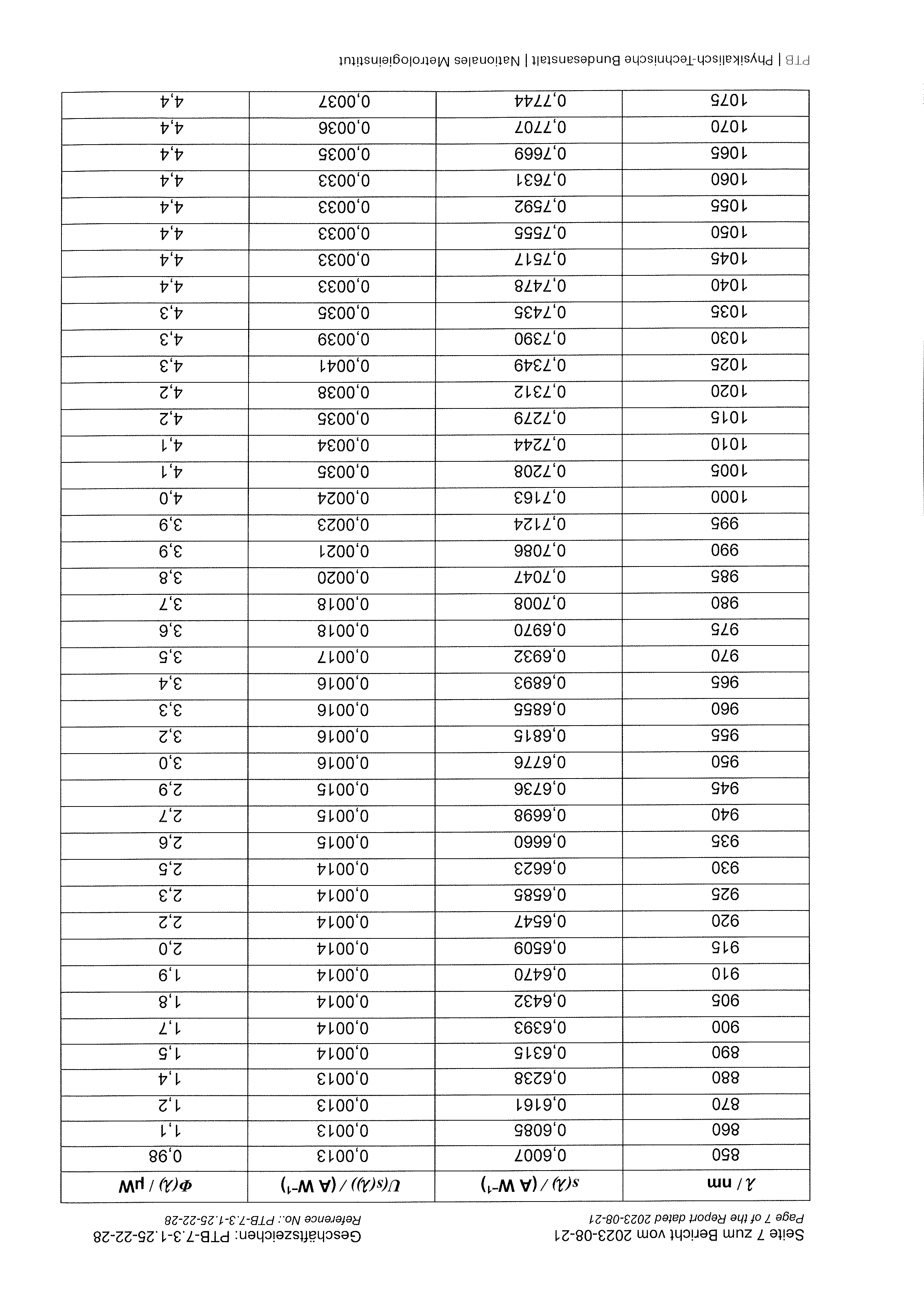


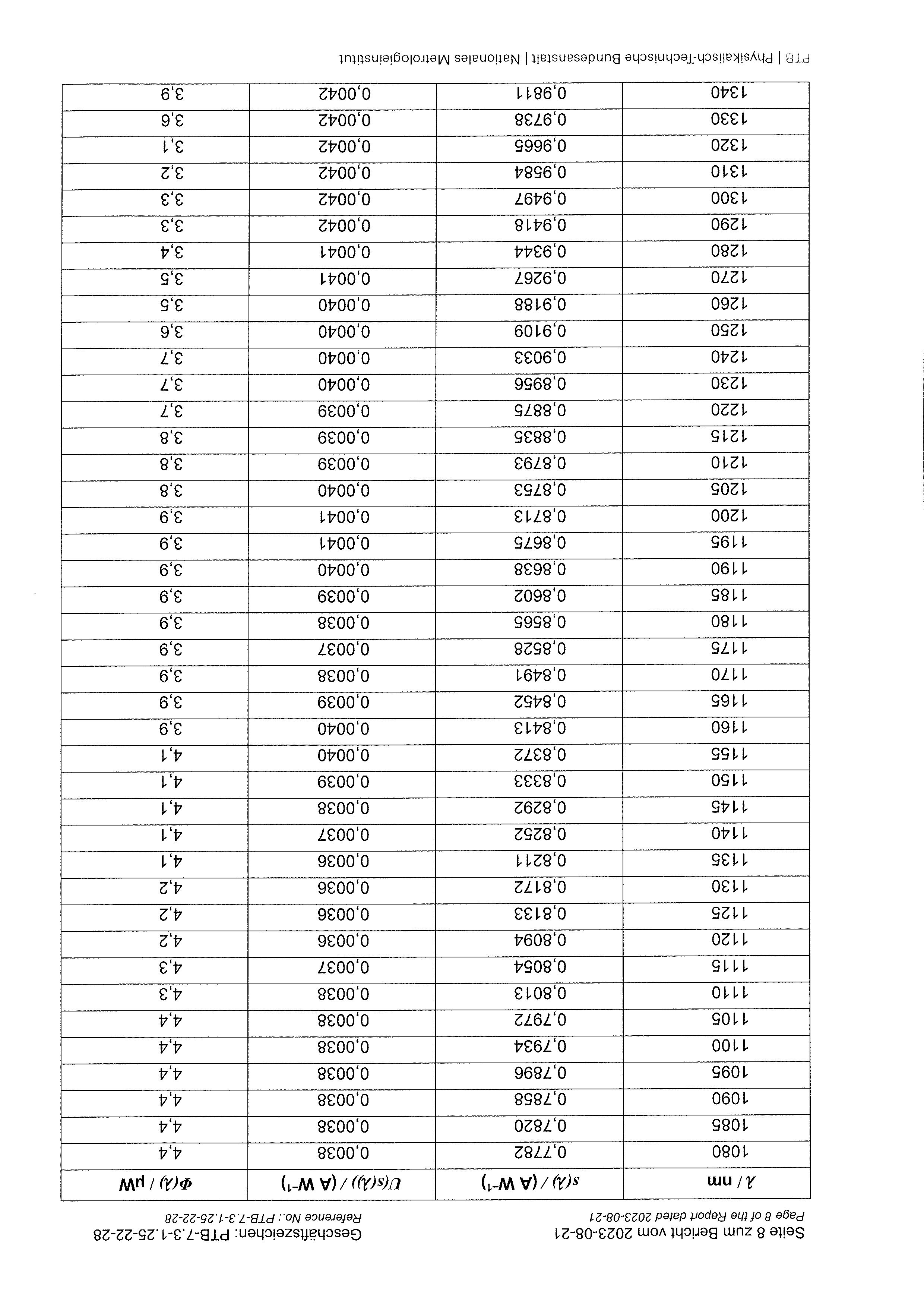


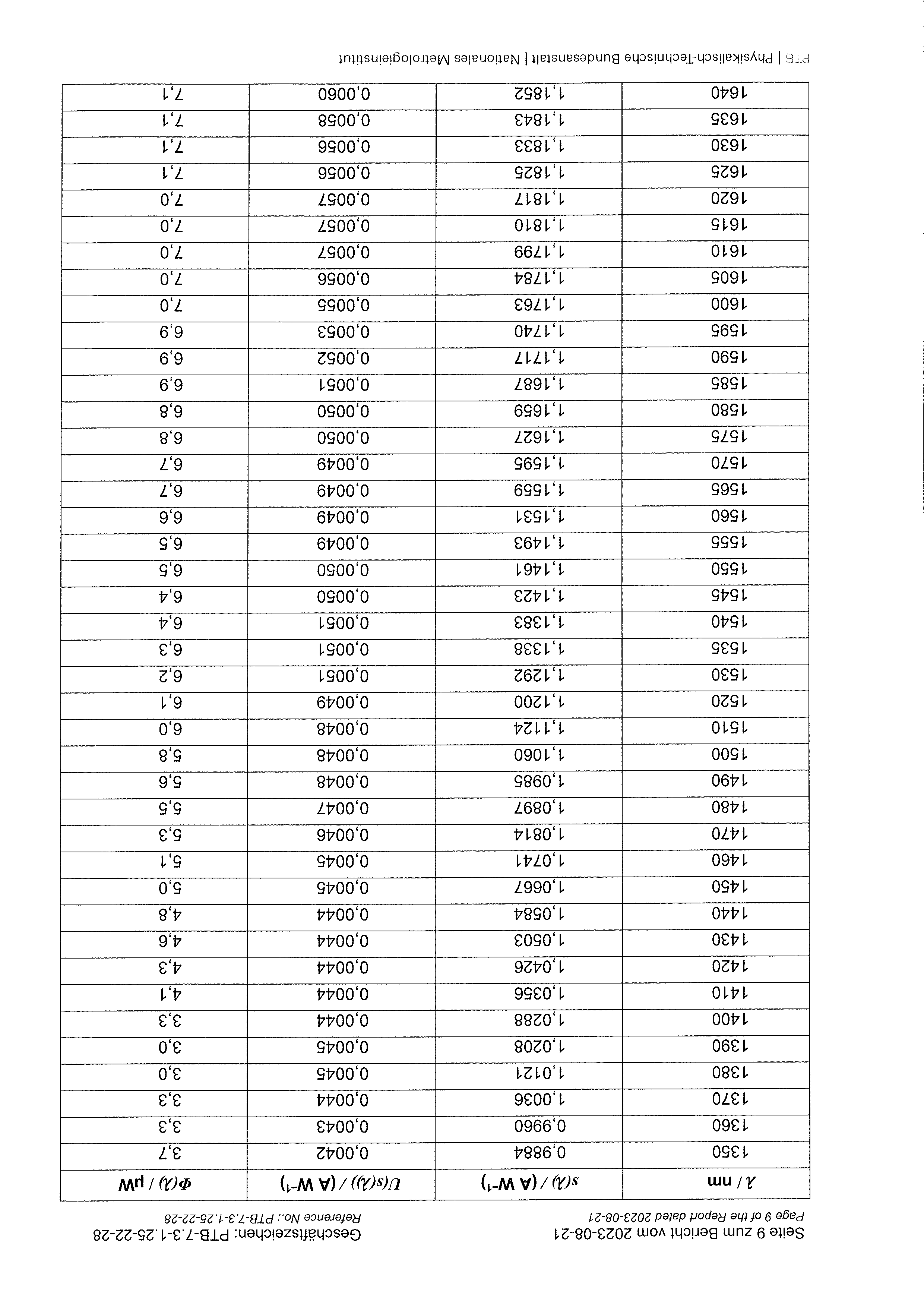


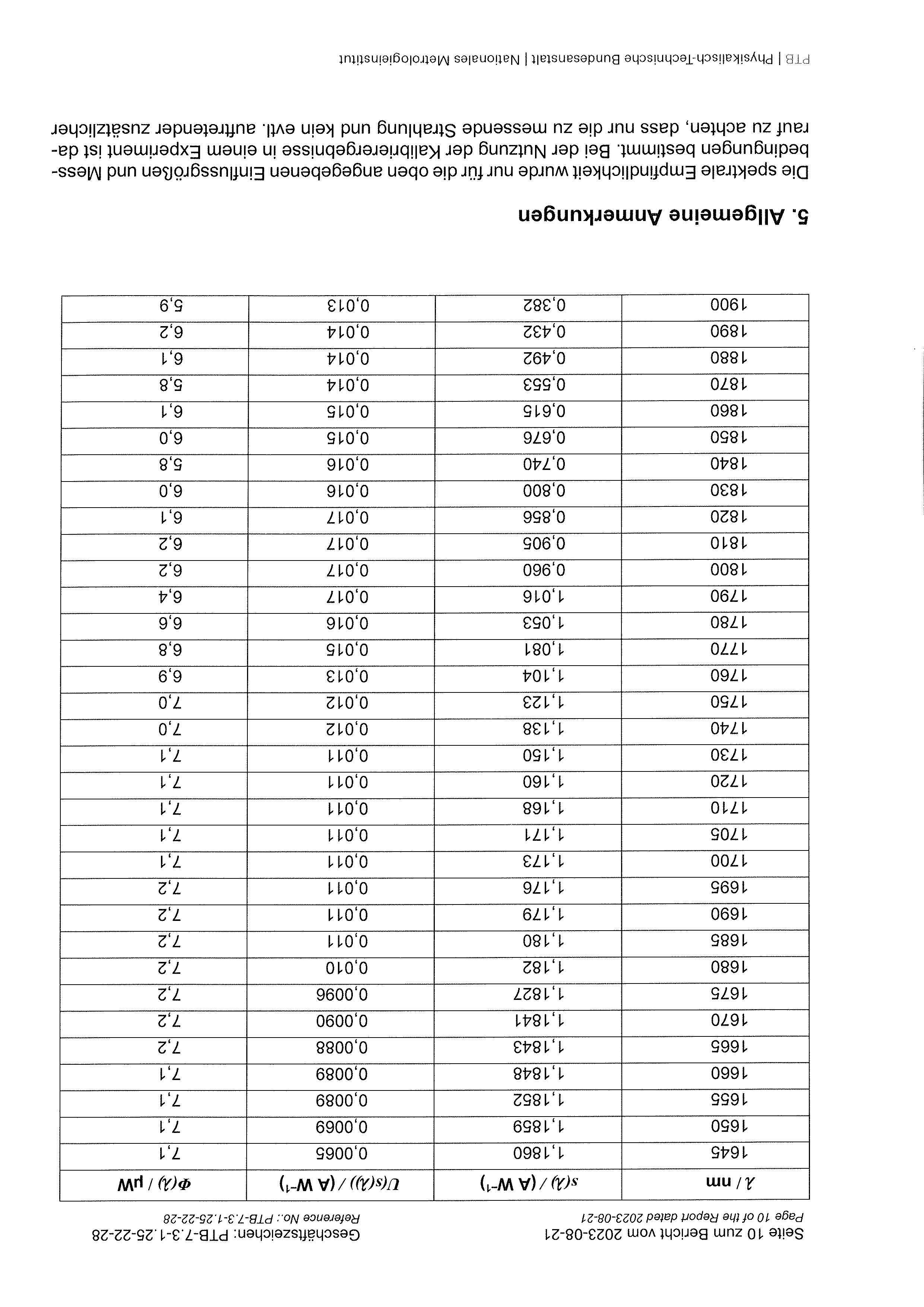


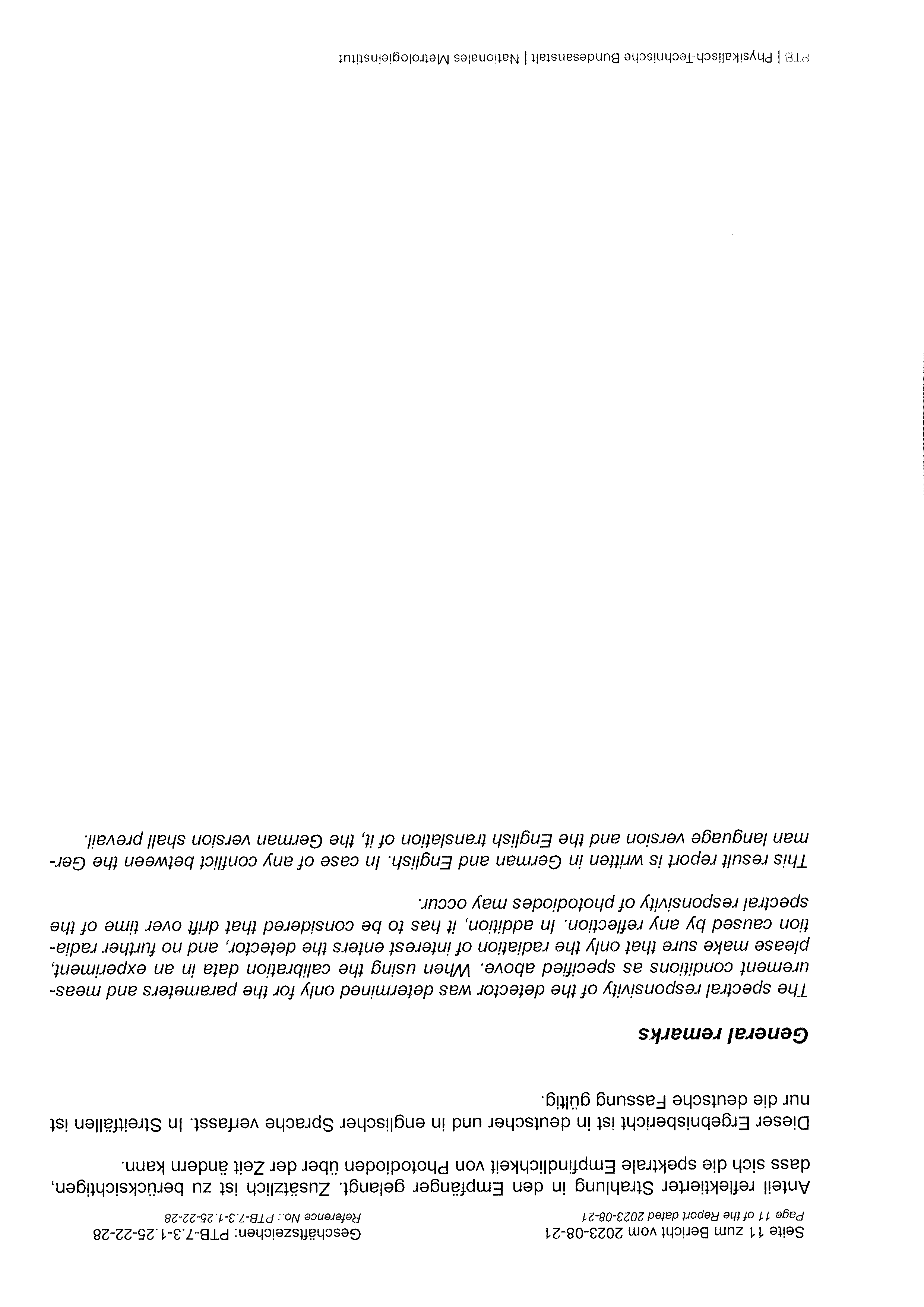


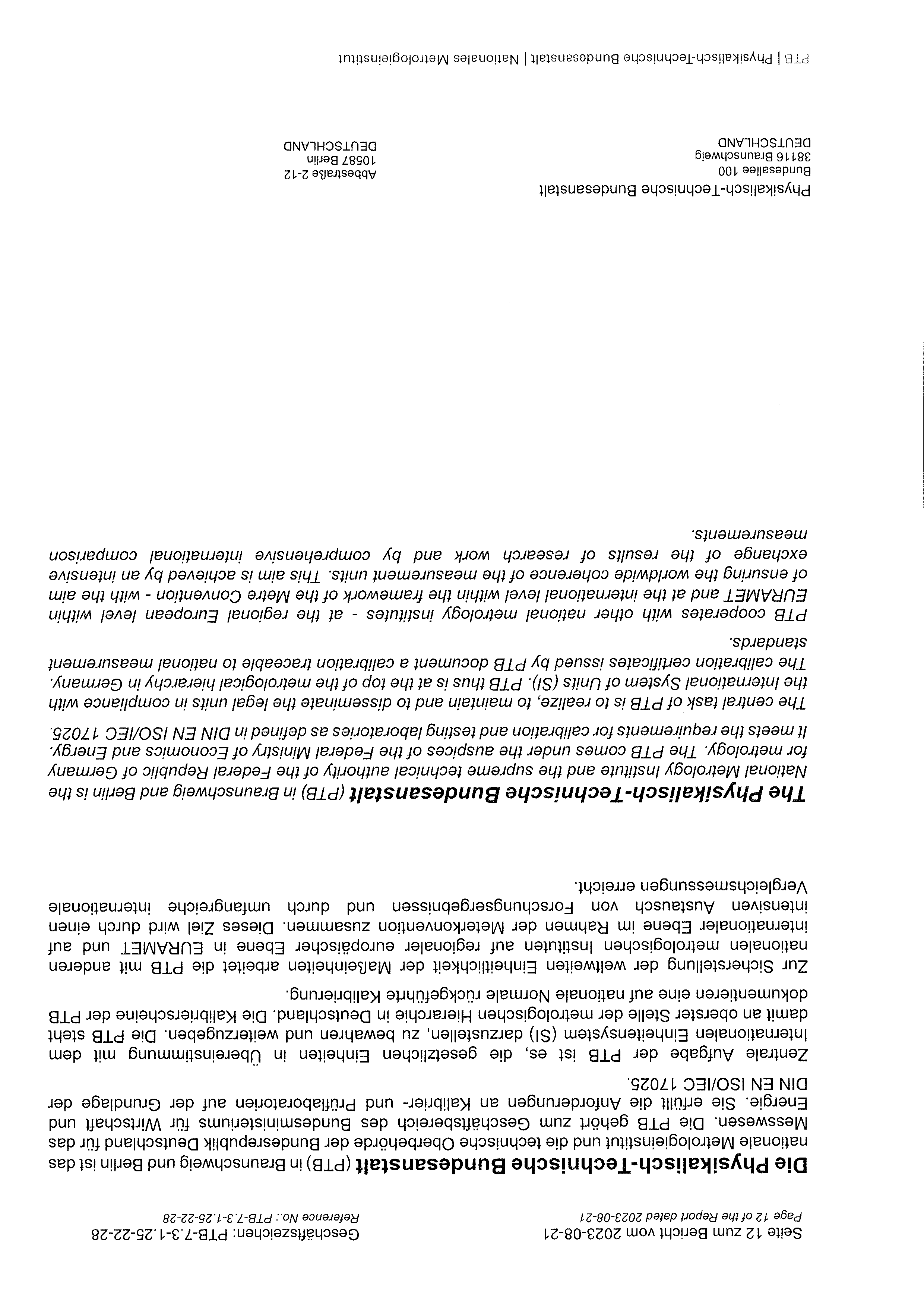

Supplement: Supplementary file 1 — Supplemental material [file 41377_2024_1670_MOESM1_ESM.docx]
